# Expanding the impact of the Driving and Dementia Roadmap through a national radio ad campaign

**DOI:** 10.1002/alz70858_101218

**Published:** 2025-12-25

**Authors:** Gary Naglie, Elaine Stasiulis, Christopher Pilieci, Mark J. Rapoport, the CCNA Driving Cessation Team Co‐Investigators and CCNA Study Group

**Affiliations:** ^1^ Baycrest Health Sciences, Toronto, ON, Canada; ^2^ University of Toronto, Toronto, ON, Canada; ^3^ Sunnybrook Health Sciences Centre, Toronto, ON, Canada

## Abstract

**Background:**

People with dementia and family and friend carers report feeling ill‐equipped to manage the driving cessation process in dementia. Our online platform, the Driving and Dementia Roadmap, provides knowledge, resources and tools to help manage the driving cessation process and provide support for when driving stops. The objective of the present study was to explore the impact of a national radio ad campaign to expand the use of the Driving and Dementia Roadmap.

**Methods:**

In January 2024, radio ads targeting people with dementia and family/friend carers were broadcasted to 5 Canadian provinces. Excluding regions with less than 4 users in January 2024, a total of 43 regions received the radio ad (coverage regions) and 39 regions did not (non‐coverage regions). Google Analytics of the Driving and Dementia Roadmap were collected in January 2024 (broadcast period) and September‐December 2023 (pre‐broadcast period). Four pre‐post outcomes were compared within‐ and between‐ regions: total users, new users, return users, and engaged sessions (i.e., visits > 10 seconds). Data were analyzed using Wilcoxon‐Signed Rank tests for within‐region comparisons and Mann‐Whitney U tests for between‐region comparisons.

**Results:**

Within both coverage and non‐coverage regions, the number of total users, new users, return users, and engaged sessions significantly increased during the broadcast period compared to pre‐broadcast (all *p*'s < .001). The pre‐post broadcast increase in total users, new users, and engaged sessions was significantly greater within the coverage regions compared to non‐coverage regions (all *p*'s < .05), with the greatest increase being in the number of total users (*p* < .001). On average, the pre‐post monthly broadcast increase in total users was four‐fold larger within the coverage regions (M=20.19; SD=57.44) compared to non‐coverage regions (M=5.22; SD=4.47).

**Conclusion:**

This national radio ad campaign had a significant impact on use of the Driving and Dementia Roadmap, increasing total users, new users, and engaged sessions. Overall, this expanded use will help more people with dementia and their family/friend carers access knowledge, resources, and tools on ways to better manage the complex process of driving cessation.

